# Occurrence, Antibiotic Resistance, and Population Diversity of *Listeria monocytogenes* Isolated From Fresh Aquatic Products in China

**DOI:** 10.3389/fmicb.2018.02215

**Published:** 2018-09-19

**Authors:** Moutong Chen, Jianheng Cheng, Qingping Wu, Jumei Zhang, Yuetao Chen, Liang Xue, Tao Lei, Haiyan Zeng, Shi Wu, Qinghua Ye, Jianling Bai, Juan Wang

**Affiliations:** ^1^State Key Laboratory of Applied Microbiology Southern China, Guangdong Provincial Key Laboratory of Microbial Culture Collection and Application, Guangdong Open Laboratory of Applied Microbiology, Guangdong Institute of Microbiology, Guangzhou, China; ^2^College of Food Science, South China Agricultural University, Guangzhou, China

**Keywords:** *Listeria monocytogenes*, multi-locus sequence typing, antibiotic resistance, aquatic products, *llsX*, cellobiose family PTS, premature stop codon

## Abstract

*Listeria monocytogenes* is an important Gram-positive foodborne pathogen. However, limited information is available on the comprehensive investigation and potential risk of *L. monocytogenes* in fresh aquatic products, which are popular to consumers in China. This study aimed to determine the occurrence, virulence profiles, and population diversity of *L. monocytogenes* isolated from aquatic products in China. In total, 846 aquatic product samples were collected between July 2011 and April 2016 from 43 cities in China. Approximately 7.92% (67/846) aquatic product samples were positive for *L. monocytogenes*, 86.57% positive samples ranged from 0.3 to 10 MPN/g, whereas 5.97% showed over 110 MPN/g by the Most Probable Number method, which included two samples of products intended to be eaten raw. Serogroups I.1 (serotype 1/2a), I.2 (serotype 1/2b), and III (serotype 4c) were the predominant serogroups isolated, whereas serogroup II.1 (serotype 4b) was detected at much lower frequencies. Examination of antibacterial resistance showed that nine antibacterial resistance profiles were exhibited in 72 isolates, a high level susceptibility of 16 tested antibiotics against *L. monocytogenes* were observed, indicating these common antibacterial agents are still effective for treating *L. monocytogenes* infection. Multilocus sequence typing revealed that ST299, ST87, and ST8 are predominant in aquatic products, indicating that the rare ST299 (serotype 4c) may have a special ecological niche in aquatic products and associated environments. Except *llsX* and *ptsA*, the 72 isolates harbor nine virulence genes (*prfA, actA, hly, plcA, plcB, iap, mpl, inlA*, and *inlB*), premature stop codons (PMSCs) in *inlA* were found in four isolates, three of which belonged to ST9. A novel PMSC was found in 2929-1LM with a nonsense mutation at position 1605 (TGG→TGA). All ST87 isolates harbored the *ptsA* gene, whereas 8 isolates (11.11%) carried the *llsX* gene, and mainly belonged to ST1, ST3, ST308, ST323, ST330, and ST619. Taken together, these results first reported potential virulent *L. monocytogenes* isolates (ST8 and ST87) were predominant in aquatic products which may have implications for public health in China. It is thus necessary to perform continuous surveillance for *L. monocytogenes* in aquatic products in China.

## Introduction

*Listeria monocytogenes* is an important foodborne pathogen worldwide, that can cause life-threatening listeriosis disease in vulnerable groups including pregnant women, fetuses, elderly people, and immunocompromised individuals, with a considerable mortality rate (20–30%) (Lomonaco et al., 2009). *L. monocytogenes* is capable of proliferating in different stressful environmental conditions, including high salinity, low temperature, and a wide range of pH values. Foodborne listeriosis poses a global economic and health burden due to the wide spread of *L. monocytogenes* in food and food processing environments.

In China, 147 clinical cases in total and 82 outbreak-related cases were reported in 28 provinces from January 1964 to December 2010 (Feng et al., 2013). In recent years, listeriosis diseases have been increasingly reported in China annually, especially in developed cities (Wang et al., 2015, 2018). This trend may be linked to the trend of direct consumption of fresh foods and ready-to-eat foods in China, especially in developed cities. However, there is no national monitoring system for listeriosis cases in China as it is not yet considered a notifiable disease. According to the report of the European Food Safety Authority (EFSA), the small number of listeriosis outbreaks may be linked to the lower consumption of fish and fish products compared to other foods in Europe (European Food Safety Authority [EFSA] and European Centre for Disease Control [ECDC], 2015). Similarly, as reported by Song et al. (2015), ready-to-eat (RTE) raw fish has the highest per serving risk of listeriosis among the five kinds of ready-to-eat foods based on risk assessment. This indicates that RTE fish and fish products may serve as a source of *L. monocytogenes* infection. Previous studies have focused on the prevalence of *L. monocytogenes* in aquatic products, but these studies only focused their investigation to limited cities/provinces, such as Jiangsu Province, Henan Province, Beijing City, and Zhanjiang City (Chen et al., 2013; Ma, 2015; Wu et al., 2017; Chui et al., 2018). However, little information is available on the comprehensive investigation and potential virulence of *L. monocytogenes* from aquatic products in China. According to data compiled by the National Bureau of Statistics of China, the average consumption of aquatic products per capita has increased annually and in 2016, it was 11.4 kg^1^ . In this context, it is necessary to systematically investigate the contamination level of *L. monocytogenes* in aquatic products in China.

*Listeria monocytogenes* has been differentiated into 13 serotypes based on its somatic (O) and flagellar (H) antigens, and are further grouped into five serovars, designated as serogroups I.1 (1/2a-3a), I.2 (1/2c-3c), II.1 (4b-4d-4e), II.2 (1/2b-3b-7), and III (4a-4c) (Doumith et al., 2004). A series of virulence factors participating in cellular infection cycles have been documented in *L. monocytogenes*. *Listeria* pathogenicity island-1 (LIPI-1, including *prfA, hly, mpl, iap, plcB, plcC*, and *actA*) and LIPI-2 (*inlA* and *inlB*) are considered as the two classic major pathogenicity islands in *L. monocytogenes*. *L. monocytogenes* is known to exhibit varied pathogenicity in intra-species isolates even though each isolate harbors both LIPI-1 and LIPI-2. In recent years, the *llsX* gene (encoding Listeriolysin, LLS, a hemolytic, and cytotoxic factor) belonging to LIPI-3, has been greatly associated with a subset of lineage I in human listeriosis (Cotter et al., 2008). Maury et al. (2016) identified that a cluster of six genes annotated as the cellobiose-family phosphotransferase system (PTS) and designated as LIPI-4, was highly associated with *L. monocytogenes* neuroinvasiveness and human maternal-neonatal (MN) infection. At present, LIPI-3 were found only in lineage I clonal complexes, 23 STs were found to carried LIPI-3 (ST1, ST1001, ST3, ST4, ST6, ST191, ST213, ST217, ST224, ST288, ST363, ST79, ST77, ST382, ST389, ST489, ST999, ST554, ST581, ST1000, ST380, ST778, and ST619), LIPI-4 presented in 11 STs belongs to lineage I and a single lineage III (ST4, ST87, ST213, ST217, ST363, ST382, ST388, ST663, ST1002, ST1166, and ST619) (Chen et al., 2018; Kim et al., 2018; Wang et al., 2018). The presence of these virulence genes may be an important determinant to their pathogenicity.

The objective of this study was to (i) comprehensively investigate the occurrence and contamination level of *L. monocytogenes* in fresh aquatic products; (ii) evaluate the potential virulence and antibacterial profiles of *L. monocytogenes* isolates; and (iii) explore the genetic diversity of *L. monocytogenes* isolates recovered from the Chinese retail aquatic system.

## Materials and Methods

### Samples

From July 2012 to April 2016, a total of 846 retail aquatic products were collected from rural markets (*n* = 245), open-air markets (*n* = 261), and large supermarkets (*n* = 340) from 43 cities of China. The marine aquatic products (*n* = 506) included squid (*n* = 150 samples), shrimp (*n* = 145), yellow croak (*n* = 100), weever (*n* = 24), shell fish (*n* = 23), saury (*n* = 20), octopus (*n* = 10), cuttlefish (*n* = 6), silvery pomfret (*n* = 3), salmon (*n* = 6), capelin (*n* = 3), and other species (*n* = 16). Freshwater aquatic products (*n* = 341) comprised the crucian carp (*n* = 117), grass carp (*n* = 82), *Tilapia mossambica* (*n* = 49), *Megalobrama amblycephala* (*n* = 29), *Cyprinus carpio* (*n* = 21), bighead carp (*n* = 7), *Pelteobagrus fulvidraco* (*n* = 5), silver carp (*n* = 5), *Channa argus* (*n* = 4), catfish (*n* = 2), and other species (*n* = 20). Except of packaged salmon products, all of marine aquatic products except shrimp were stored loosely in ice, while freshwater aquatic products were alive keeping in water. Among these samples, salmon, grass carp, and *Tilapia mossambica* were intended to be eaten raw. All samples were placed in insulated shipping coolers containing frozen gel packs, which were placed on the sides, middle, and the top of the samples. All samples were kept below 4°C during transportation and testing was initiated within 4 h after receiving the samples.

### Qualitative and Quantitative Analysis

Qualitative detection of *L. monocytogenes* was performed according to the National Food Safety Standard of China (4789.30-2010) with minor adaptations Ministry of Health of People’s Republic of China (2010). Briefly, 25 g of homogenized aquatic samples were added to 225 mL *Listeria* enrichment broth 1 (LB1) (Guangdong Huankai Co., Ltd., Guangzhou, China). The cultures in LB1 media were incubated at 30°C for 24 h, after which 0.1 mL LB1 enrichment culture was transferred to 10 mL *Listeria* enrichment broth 2 (LB2) at 30°C for 24 h. A loopful (about 10 μL) of the LB2 enrichment culture was streaked onto *Listeria* selective agar plates (Guangdong Huankai Co., Ltd.) and incubated at 37°C for 48 h. Three to five presumptive colonies were selected for the identification of *L. monocytogenes* using the Microgen ID *Listeria* identification system (Microgen, Camberley, United Kingdom) according to the manufacturer’s instructions.

The workflow for most probable number (MPN) was adapted according to a previous study by Gombas et al. (2003). Briefly, a 9-tube MPN method was used. The nine tubes were divided into three sets of three tubes. The first set of tubes was contained 10 mL of the sample homogenate, the second and third sets of tubes contained 10 mL of Fraser broth (Guangdong Huankai Co., Ltd., Guangzhou, China) inoculated with 1 and 0.1 mL of the homogenate, respectively. Three aliquots (10, 1, and 0.1 mL) of the sample homogenate were dispensed into three sets, representing 1.0, 0.1, and 0.01 g of the original sample, respectively. The tubes were incubated at 30 ± 2°C for 24 ± 2 h. Darkened Fraser tubes were subjected to streaking onto *Listeria* selective agar plates. If a Fraser broth tube did not darken, it was examined again after an additional 26 ± 2 h of incubation. The MPN value was determined based on the number of positive tube(s) in each of the three sets and the MPN table (U.S. Department of Agriculture, 1998).

### Serogroup Analysis

Genomic DNA was extracted from *L. monocytogenes* using a Bacterial Genomic DNA Purification Kit (Magen Biotech Inc., Guangzhou, China) according to the manufacturer’s instructions. Serogroup analysis of 72 isolates was performed using multiplex PCR as described by Doumith et al. (2004) (**Supplementary Table S1**). Five distinct serogroups, I.1 (1/2a-3a), I.2 (1/2c-3c), II.1 (4b-4d-4e), II.2 (1/2b-3b-7), and III (4a-4c), were identified using multiplex PCR. The primers used are shown in **Supplementary Table S2**. PCR was performed with an initial denaturation step at 94°C for 3 min; 35 cycles of 94°C for 35 s, 53°C for 50 s, and 72°C for 60 s; and a final cycle of 72°C for 7 min in a thermocycler (Applied Biosystems, CA, United States). The amplicons (8 μl) were separated on 2% agarose gels in TAE buffer and then visualized by Goldview^®^ staining (0.005%, v/v). All strains were serotyped by antigen serum agglutination according to the manufacturer’s instruction (Denka Seiken Co., Ltd., Tokyo, Japan).

### Antimicrobial Susceptibility Test

All strains collected were analyzed by the KB method using breakpoints recommended by the National Committee for Clinical Laboratory Standards (Clinical and Laboratory Standards Institute, 2014) for *Staphylococcus*, except for ampicillin and penicillin G where specific *Listeria* breakpoints are defined (M45-A2 Vol. 30 No. 18) (**Supplementary Table S3**). In total, 16 antibiotic agents, including those used to treat human listeriosis, were tested by the KB method as follows: ampicillin (AMP; 10 μg), chloramphenicol (C; 30 μg), erythromycin (E; 15 μg), gentamicin (CN; 10 μg), kanamycin (K; 30 μg), rifampin (RD; 5 μg), doxycycline (DO, 30 μg), penicillin (P, 10 U), tetracycline (TE; 30 μg), vancomycin (VA; 30 μg), sulfamethoxazole with trimethoprim (SXT; 23.75/1.25 μg), sulbactam/ampicillin (SAM; 10/10 μg), meropenem (MEM; 10 μg), linezolid (LZD, 30 μg), and amoxycillin/clavulanic acid (AMC; 10 μg) (Oxoid, Basingstoke, United Kingdom). *Staphylococcus aureus* ATCC 25923 and *Escherichia coli* ATCC 25922 were used as quality control strains. Zones of inhibition were measured with precision calipers to the nearest 0.01 mm. Isolates exhibiting resistance to at least three classes of the antimicrobial agents tested, were considered multidrug-resistant strains.

### Identification of Potential Hypervirulent Isolates

To evaluate the potential virulence of 72 *L. monocytogenes* isolates, the presence of virulence factors associated with infection cycle and invasiveness was detected by PCR. As shown in **Supplementary Table S4**, nine virulence factor genes belonging to LIPI-1 and LIPI-2 were detected by PCR. In addition, the presence of LIPI-3 and LIPI-4 genes (*llsX* and *ptsaA*, respectively) was also observed by PCR (Clayton et al., 2011; Maury et al., 2016). The premature stop codons (PMSCs) of *inlA* were determined by amplicon sequencing. The complete length of *inlA* (2403 bp) was sequenced in 72 isolates. External primers were used to amplify the complete *inlA* gene and internal primers were used for sequencing (**Supplementary Table S3**; Wu et al., 2016). The *inlA* sequences were assembled using DNAMAN software (version 8th). By comparing the obtained complete *inlA* sequence to that of the *L. monocytogenes* EGDe (Glaser et al., 2001), PMSC types were determined according to the site of PMSC-mutation in *inlA* gene as documented by Gelbicova et al. (2015).

### Multilocus Sequence Typing

MLST analysis of *L. monocytogenes* was previously reported by Ragon et al. (2008) (**Supplementary Table S5**). Briefly, each 50 μL PCR contained 5.0 μL 10× PCR buffer (TAKARA, Dalian, China), 1.5 mM MgCl_2_, 0.2 mM of each dNTP, 0.4 mM of each primer, 1.25 U Taq polymerase, and 1 μL genomic DNA. PCR was performed using the following program: 3 min initial denaturation at 94°C and 35 cycles consisting of denaturation at 94°C for 30 s, annealing at 52°C (45°C for *bglA)* for 1 min and elongation at 72°C for 2 min, followed by a final elongation for 10 min at 72°C. The PCR products were purified and sequenced by Invitrogen (Thermo Fisher, Shanghai, China). An allele number was given according to each variant locus of each housekeeping gene; sequence types (STs) and clonal complexes (CCs) were assigned *via* the *Listeria* MLST database at the Pasteur Institute website^2^. A neighbor-joining tree of *L. monocytogenes* based on the MLST of seven housekeeping genes was constructed using MEGA 7.0 (Kumar et al., 2016) with 1000 bootstrap replications. Simpson’s indexes of discrimination (DI) for MLST were calculated to determine the ability of the MLST typing method according to a previous study reported by Hunter and Gaston (1988).

### Data Analysis

The statistically significant difference of prevalence of *L. monocytogenes* between marine aquatic products and freshwater aquatic products was calculated using the Chi-square test. *P*-values of <0.05 were considered statistically significant.

## Results

### Occurrence and Contamination Level of *L. monocytogenes*

**FIGURE 1:**
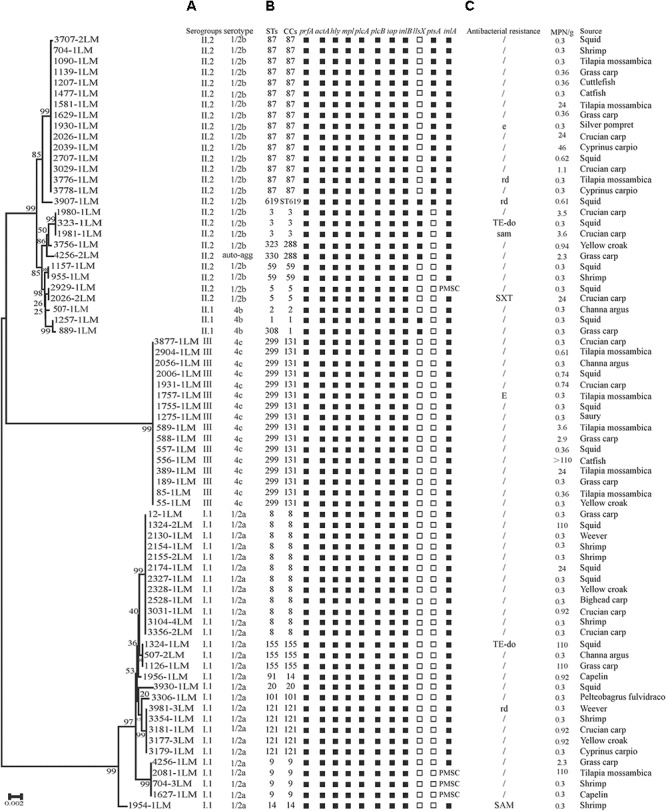
Genotypic and phenotypic characteristics of *Listeria monocytogenes* isolated from aquatic products in China. **(A)** Auto-agg: auto-aggregation. **(B)** PMSC, premature stop codons in *inlA*; solid squares indicate the presence of virulence genes or full-length *inlA*. Hollow squares indicate the absence of virulence genes. **(C)** E(e), erythromycin; DO(do), doxycycline; SXT(sxt), sulfamethoxazole with trimethoprim; OFX(ofx), ofloxacin; RD(rd), rifampin; SAM(sam), sulbactam/ampicillin; TE, tetracycline. A slash (/) indicates no resistance. Antibiotic abbreviations in uppercase indicate resistance, while those in lowercase indicate intermediate resistance. A neighbor-joining tree of *L. monocytogenes* based on the MLST of seven housekeeping genes was established in MEGA 7.0 with 1000 bootstrap replications. Bootstrap values are shown at the nodes.

The overall prevalence of *L. monocytogenes* in freshwater and marine products tested during 2012–2016 is presented in **Table 1**. In total, 67 (7.92%) samples were positive for *L. monocytogenes* among 846 analyzed samples, 28 (8.24%) positive samples from large supermarket (*n* = 340), 25 (9.58%) samples from open-air markets (*n* = 261), 14 (5.71%) samples from rural markets (*n* = 245). Among 506 marine aquatic product and 340 freshwater aquatic product samples, the prevalence of *L. monocytogenes* in freshwater aquatic products (10.29%) was found to be significantly higher than that (6.32%) in marine aquatic products (*p* < 0.05). The contamination level by *L. monocytogenes* in aquatic products was determined by the MPN method. Approximately 86.57% of positive samples ranged from 0.3 to 10 MPN/g, five positive samples were ranged from 10 to 110 MPN/g, of which one sample from marine products and four samples from freshwater products, only four (5.97%) samples were over 110 MPN/g, including one sample from marine products and three samples from freshwater products. In addition, two of the samples with *L. monocytogenes* higher than 100 MPN/g, namely grass carp and *Tilapia mossambica* were intend to be eaten raw (**Table 2** and **Figure 1**).

**Table 1:** Occurrence and serogroups of *Listeria monocytogenes* identified in this study.

Sources of samples	No.(%) of samples		Serogroups (%)			
		
	Tested	Positive for *L. monocytogenes*	I.1 (1/2a-3a)	II.1 (4b-4d-4e)	II.2 (1/2b-3b-7)	III (4a-4c)
**Marine aquatic products**						
Squid	150	14 (9.33)	5	1	6	3
Shrimp	145	7 (4.83)	6		2	
Yellow croaker	100	4 (4.00)	2		1	1
Weever	24	2 (8.33)	2			
Saury	20	1 (5.00)				1
Octopus	10	0				
Cuttlefish	6	1 (16.67)			1	
Silvery pomfret	6	1 (16.67)			1	
Salmon	3	0				
Capelin	3	2 (66.67)	2			
Oyster	23	0				
Others	16	0				
Subtotal	506	32 (6.32)	17	1	11	5
**Freshwater aquatic products**						
Crucian carp	117	9 (7.69)	4		4	2
Grass carp	83	8 (9.64)	3	1	4	2
*Tilapia mossambica*	49	9 (18.37)			3	5
*Megalobrama amblycephala*	29	0				
Bighead carp	7	1 (14.29)	1			
*Cyprinus carpio*	21	3 (14.29)	1		2	
*Pelteobagrus fulvidraco*	5	1 (20.00)	1			
Silver carp	5	0				
Catfish	5	2 (40.00)			1	1
Channa argus	5	2 (40.00)	1	1		1
Others	14	0				
Subtotal	340	35 (10.29)	11	2	14	11
Total	846	67 (7.92)	28 (38.89)	3 (4.17)	25 (34.72)	16 (22.22)


**Table 2:** Contamination levels of *Listeria monocytogenes* in aquatic products.

Sources of samples	0.3 ≤ MPN < 10 (%)	10 ≤ MPN < 110 (%)	MPN ≥ 110 (%)	Total
Marine aquatic products	30 (93.75)	1 (3.13)	1 (3.12)	32
Freshwater aquatic products	28 (80.00)	4 (11.43)	3 (8.57)	35
Total	58 (86.57)	5 (7.46)	4 (5.97)	67


### Serogroups

As shown in **Table 1**, four serogroups were identified among 72 *L. monocytogenes* isolates. Serogroup I.1 (1/2a-3a) (*n* = 28, 38.89%) was the most prevalent, followed by serogroup II.2 (1/2b-3b-7) (*n* = 25, 34.72%), and three isolates (4.17%) were identified as II.1 (4b-4d-4e). Sixteen (22.22%) isolates belonged to serogroup III (4a-4c), including four isolates from marine products and 12 isolates from freshwater products. All 72 strains were serotyped by antigen serum agglutination. Except for auto-aggregation of 4256-1LM isolate, 24 isolates were typed as 1/2b, 3 isolates as 4b, 16 isolates as 4c, and 28 isolates as 1/2a.

### Presence of Virulence Genes

The presence of 11 known virulence genes was identified in the 72 isolates in this study by PCR. As shown in **Figure 1**, except *llsX* and *ptsA*, all 72 isolates were positive for nine virulence genes, including *prfA, actA, hly, iap, mpl, plcA, plcB, inlA*, and *inlB*. Eight isolates (11.11%) carried *llsX* and mainly belonged to ST1 (*n* = 1), ST3 (*n* = 3), ST308 (*n* = 1), ST323 (*n* = 1), ST330 (*n* = 1), and ST619 (*n* = 1). *pstaA* was harbored in 16 isolates, of which 15 isolates were from ST87 and one isolate belonged to ST619. *pstaA* was absent in the other isolates. Furthermore, the PMSCs in *inlA* were analyzed by sequencing. Compared to the full-length sequence of *inlA* in the reference *L. monocytogenes* EGDe (Glaser et al., 2001), a total of 68 isolates (94.44%) carried the complete codon sequence of *inlA*, including three isolates belonging to ST9 and one ST5 isolate. As shown in **Figure 1**, four isolates (1627-1LM, 2081-1LM, 704-3LM, and 2929-1LM) were found to carry PMSCs in *inlA*. In the isolate 1627-1LM, sequence analysis revealed a nonsense mutation at position 2054, which resulted in changing a glutamine codon to a stop codon (TGG→TAG). In 2081-1LM, a nonsense mutation at position 976 changed a glutamic acid (Glu) codon to a stop codon (GAA→TAA). Adenylic acid deletion at position 12 resulted in a PMSC in *inlA* of 704-3LM. A nonsense mutation was found at position 1605 (TGG→TGA) in the 2929-1LM isolate, and was identified as a novel type of point mutation in *inlA* based on the previously known PMSC mutation types of *inlA* (Gelbicova et al., 2015; Moura et al., 2016; **Table 3**).

**Table 3:** Premature stop codons identified in inlA gene of *Listeria monocytogenes*.

PMSC type	Nucleotide position of mutation	Length of truncated InlA	Strain number	Reference
1	1818 (T→A)	605	FSL F2-563	Nightingale et al., 2005
2	1966 (C→T)	655	FSL R2-074	Nightingale et al., 2005
3	2100 (C→G)	699	FSL F2-516	Nightingale et al., 2005
4	12 (deletion A)	8	F7-061	Felicio et al., 2007
5	565 (C→T)	188	FSL R2-080	Van Stelten and Nightingale, 2008
6	1474 (C→T)	491	H1	Olier et al., 2003
7	1684 (C→T)	561	FSL T1-061	Van Stelten and Nightingale, 2008
8	1380 (G→A)	459	NV8	Rousseaux et al., 2004
9	1540 (deletion G)	518	NV7	Rousseaux et al., 2004
10	1961 (insertion T)	676	NV4	Rousseaux et al., 2004
11	2054 (G→A)	684	NV5	Rousseaux et al., 2004
12	1637 (deletion A)	576	LO28	Jonquieres et al., 1998
13	1579 (A→T)	526	36-25-1	Handa-Miya et al., 2007
14	1615 (C→T)	538	LM57179	Ragon et al., 2008
15	229 (C→T)	76	NRRL_B-57040	Van Stelten et al., 2010
16	508 (G→T)	169	NRRL_B-33873	Van Stelten et al., 2010
17	758 (T→A)	252	NRRL_B-57066	Van Stelten et al., 2010
18	1165 (deletion T)	403	NRRL_B-33591	Van Stelten et al., 2010
19	976 (G→T)	325	L3102	Gelbicova et al., 2015
Novel	1605 (G→A)	535	2929-1LM	This study


### Antibacterial Susceptibility Test

**FIGURE 2:**
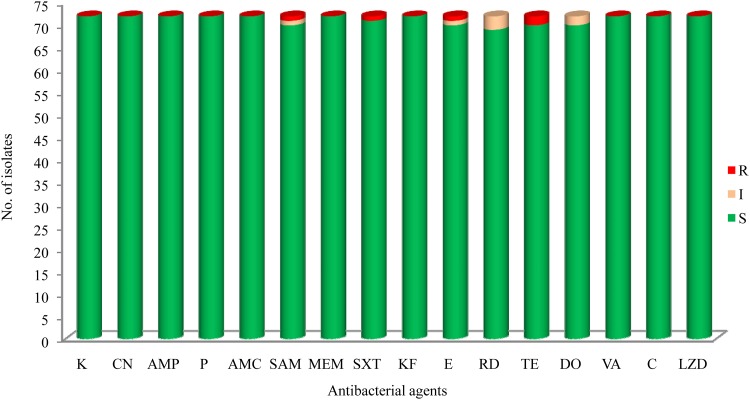
The results of common antibacterial agents against *Listeria monocytogenes* isolates^∗^. ^∗^C, chloramphenicol; K, kanamycin; CN, gentamicin; LEV, levofloxacin; OFX, ofloxacin; CIP, ciprofloxacin; AMP, ampicillin; P, penicillin; AMC, amoxycillin/clavulanic acid; SAM, sulbactam/ampicillin; MEM, meropenem; SXT, sulfamethoxazole with trimethoprim; KF, cephalothin; E, erythromycin; RD, rifampin; TE, tetracycline; DO, doxycycline; VA, vancomycin; LNZ, linezolid.

The results of antibacterial susceptibility of 72 isolates against 16 antibacterial agents are shown in **Figure 2**. Nine antibacterial resistance profiles were found in the 72 *L. monocytogenes* isolates. The number of antibiotic-resistant *L. monocytogenes* isolates was relatively low; most isolates were susceptible to the 16 tested antibacterial agents. None of the isolates exhibited resistance to nine antibacterial agents, which are ampicillin, penicillin, kanamycin, genmycin, amoxycillin/clavulanic acid, meropenem, vancomycin, chloramphenicol, and linezolid. None isolate was considered as multidrug resistant isolates, and were resistant to three classes of tested antibacterial agents (**Figure 1**).

### MLST Analysis

**FIGURE 3:**
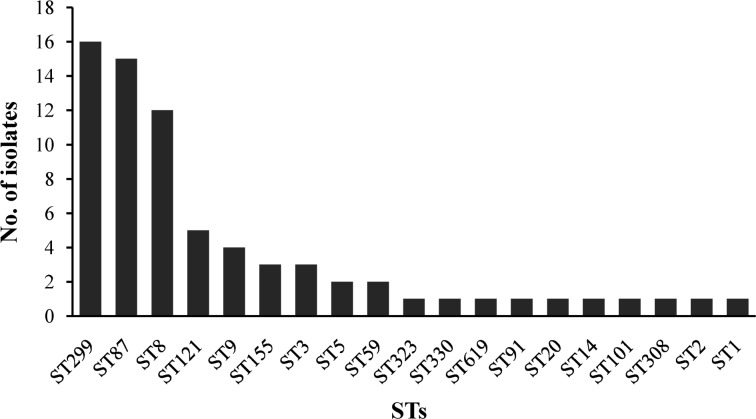
The ST distributions of *Listeria monocytogenes* isolates by MLST analysis.

The 72 isolates were distributed into 19 different STs belonging to 16 complex clones (CCs), with a DI of 0.877. Ten STs that contained between 2 and 16 isolates and 9 singletons were included in the 19 STs. Of the major STs containing at least 12 isolates (ST299, ST87, and ST8), ST299 (*n* = 16, 22.22%) was the most prevalent, followed by ST87 (*n* = 15, 20.83%), and ST8 (*n* = 12, 16.67%). The top three STs (ST299, ST87, and ST8) comprised 59.72% of all isolates (**Figure 3**).

## Discussion

*Listeria monocytogenes*, an important human foodborne pathogen, is of great public health concern. Because of its ability to resist various stress environments in food processing, the food chain is considered the main transmission route of *L. monocytogenes* to consumers. Fresh aquatic products are a popular food in China because of their taste and nutritional value. Some countries have formulated a safety criterion of <100 CFU/g for fishery products (including smoked fish) (Anonymous, 2005; International Commission on Microbiological Specifications for Foods [ICMSF], 2011), there are no qualitative and quantitative criteria or regulations for *L. monocytogenes* in fresh fish products in China. It is therefore necessary to explore the potential risk of *L. monocytogenes* infection upon consumption of fresh fish in China. However, little information on comprehensive investigation of *L. monocytogenes* contamination in fresh aquatic products in China was available. This study thus aimed to determine the occurrence, antibacterial susceptibility, and genotypic characteristics of *L. monocytogenes* isolates recovered from fresh aquatic products in the Chinese food system.

In this study, 67 samples (7.92%) out of 846 fresh aquatic products were positive for *L. monocytogenes*. This is in agreement with the results of a study conducted in Beijing City (Ma, 2015), whereas several previous studies reported a lower prevalence of *L. monocytogenes* in different cities in China, ranging from 1.57 to 4.88% (Chen et al., 2013; Wu et al., 2017; Chui et al., 2018). These discrepancies may be due to different sample composition of aquatic products and/or the difference farming, storage and processing of aquatic products. In addition, the prevalence of *L. monocytogenes* in freshwater aquatic products was found to be significantly higher than that in marine aquatic products. Quantitative analysis also showed that only four positive samples were over 110 MPN/g, of which one was a marine fish sample and three were freshwater aquatic products, two samples (*Tilapia mossambica*, and grass carp) were intended to be eaten by raw, indicating potential risk was present in aquatic products. In addition, these results indicate that contamination with this pathogen may be higher in freshwater fish, which may be associated with the different storage temperature between marine aquatic products and freshwater aquatic products. This result is consistent with the results of Wieczorek and Osek (2017).

In recent years, the emerging prevalence of antibiotic resistance in *L. monocytogenes* has been reported in Asia as well as globally (Pagadala et al., 2012; Chen et al., 2015; Obaidat et al., 2015; Sharma et al., 2017; Akrami-Mohajeri et al., 2018; Wilson et al., 2018). The findings of this study suggested that the prevalence of antibiotic resistance among *L. monocytogenes* isolated from fish products in China is relatively low (**Figure 2**). Notably, unlike the high susceptibility of ampicillin and penicillin against *L. monocytogenes* in this study, a high level of resistance was also reported in ready-to-eat foods in Gondar Town, Ethiopia (66.7%) (Garedew et al., 2015), dairy-based food products in Lebanon (90.0%) (Harakeh et al., 2009), milk and dairy products in Yazd, central Iran (Akrami-Mohajeri et al., 2018), and retail foods in Turkey (72.2%) (Cetinkaya et al., 2014). Penicillin is the first-line antibiotic therapy alone or with gentamicin for treating *L. monocytogenes* infection, but sulfamethoxazole and trimethoprim is an optional choice for penicillin-allergic patients, and erythromycin is used in pregnant women. In this study, we found a high level of susceptibility to common antibiotics including gentamicin, sulfamethoxazole and trimethoprim, and erythromycin, against *L. monocytogenes* isolates, which is consistent with previous studies (Shen et al., 2013; Chen et al., 2014, 2015; Wieczorek and Osek, 2017). Although our data are limited, antibiotic-resistant *L. monocytogenes* isolates are relatively low in this study (**Figure 1**), further studies are required to surveil the antibiotic susceptibility of foodborne *L. monocytogenes*.

The pathogenicity of *L. monocytogenes* is known to differ at an intra-species level. Serotype 4b, 1/2a, and 1/2b account for 95% of the strains isolated from human listeriosis (Cartwright et al., 2013; Althaus et al., 2014). In this study, the majority of *L. monocytogenes* belonged to serogroup I.1 (serotype 1/2a, 38.89%), II.2 (serotype 1/2b, 34.72%), and III (serotype 4c, 22.22%), which is consistent with the previous results obtained in fish from several countries (Fallah et al., 2013; Jamali et al., 2015; Terentjeva et al., 2015). Wang et al. (2015) reported serotype 1/2b (belonged to ST87 and ST3) with a frequency of 64.3% of *L. monocytogenes* was predominant in human listeriosis cases in China, similar results was also reported in Zigong, Sichuan province, conducted by Wang et al. (2018), indicating potential hypervirulent isolates were present in aquatic products in China. The three most common STs were ST299, ST87, and ST8 in this study, Wang et al. (2012) reported ST9 (29.1%), ST8 (11.7%), and ST87 (10.7%) of *L. monocytogenes* predominated in animal foods in China, similar results of ST9 (72%) was found in raw pork and associated environments in China (Li et al., 2018), ST2 was predominated in some Brazilian dairy industries and retail products. Serogroup III (4a-4c) was mainly isolated from ruminants (Orsi et al., 2011), and 22.22% of isolates in this study were identified as serogroup III (ST299), which is in agreement with previous observations in Beijing, China (Ma, 2015). These results indicating that the rare ST299 (serotype 4c) may have a special ecological niche in aquatic products and associated environments, such as aquacultural water, containers, and chilling rooms, in China. Therefore, a comprehensive study is needed to explore whether ST299 (serotype 4c) were predominant in aquatic products and associated environments. To date, several well-known virulence genes of *L. monocytogenes* have been documented in previous studies, and have been used for assessing potential risk, by detecting the presence of these genes. In the present study, the 72 isolates harbored the nine classical virulence genes belonging to LIPI-1 and LIPI-2, including *prfA, hly, actA, plcA, plcB, mpl, iap, inlA*, and *inlB*. However, four isolates showed PMSCs in *inlA*, which significantly attenuates the ability to invade host cells (Nightingale et al., 2008; Van Stelten et al., 2010). According to the PMSC type of *inlA* reported in previous studies (Gelbicova et al., 2015; Moura et al., 2016), a novel type of PMSC in *inlA* was found at position 1605 (TGG→TGA) in isolate 2929-1LM. Interestingly, three fourths of isolates with PMSCs belonged to ST9 in this study. In contrast, none of the ST121 isolates was identified as PMSC, which was most identified as PMSCs in food and associated environments in the other countries (Hein et al., 2011; Ciolacu et al., 2015; Palma et al., 2017). In recent years, LIPI-3 genes were identified as important virulence factors that acts to target the host gut microbiota, responsible for the majority of listeriosis outbreaks (Cotter et al., 2008; Quereda et al., 2017), and human central nervous system (CNS) or MN listeriosis was mainly attributed to LIPI-4 (Maury et al., 2016). Eight isolates (11.11%) carried the *llsX* gene and mainly belonged to ST1, ST3, ST308, ST323, ST330, and ST619, whereas 15 isolates belonging to ST87 showed the presence of LIPI-4 in this study. LIPI-3 were mainly present in lineage I, included 21 STs belongs to 18 CCs (CC1, CC3, CC4, CC6, CC191, CC213, CC217, CC224, CC288, CC363, CC379, CC382 CC389, CC489, CC554, CC581, CC1000, and CC380) (Chen et al., 2018; Hilliard et al., 2018; Kim et al., 2018; Wang et al., 2018). While LIPI-4 were only reported in CC4 isolates by Maury et al. (2016), recent studies reported that LIPI-4 presented in several other lineage I CCs and in a single lineage III CC in China, the United States, and Ireland, namely ST4, ST87, ST213, ST217, ST363, ST382, ST388, ST663, ST1002, ST1166, and ST619 (Chen et al., 2018; Hilliard et al., 2018; Kim et al., 2018; Wang et al., 2018). Importantly, isolate 3907LM belonging to ST619 carried both LIPI-3 and LIPI-4. Isolates belonging to ST8/CC8 are also potential hypervirulent to humans, and are known to cause listeriosis in China as well as other countries (Althaus et al., 2014; Jensen et al., 2016; Oxaran et al., 2017; Wang et al., 2018). Unlike western countries, in which the majority of clinical isolates mainly belong to CC1, CC2, CC6, CC4, and CC101, which have been demonstrated to be strongly associated with human listeriosis (Maury et al., 2016; Hilliard et al., 2018; Kim et al., 2018), CC87 is predominant in human listeriosis in China (Huang et al., 2015; Wang et al., 2018). These results demonstrate that potential virulent strains isolated from aquatic products pose a potential health risk to consumers in China.

## Conclusion

In conclusion, *L. monocytogenes* is an important foodborne pathogen worldwide. Of fresh aquatic products samples, which are popular among consumers in China, approximately 7.92% were positive for *L. monocytogenes*. Of these, 86.57% positive samples ranged from 0.3 to 10 MPN/g and 5.97% showed over 110 MPN/g by the MPN method, two samples (*Tilapia mossambica*, and grass carp) were intended to be eaten by raw. Serogroup I.1 (serotype 1/2a), I.2 (serotype 1/2b), and III (serotype 4c) were predominant in aquatic products and most of the common antibacterial agents are still effective for treating *L. monocytogenes* infection. The three most common STs were ST299, ST87, and ST8, indicating that the rare ST299 (serotype 4a, 4c) may have a special ecological niche in aquatic products and associated environments in China. PMSCs in *inlA* were found in four isolates, of which three belonged to ST9. A novel PMSC at position 1605 (TGG→TGA) in *inlA* was found in 2929-1LM. Several STs (ST87, ST8, ST1, ST3, ST308, ST323, ST330, and ST619) found in aquatic products have been previously reported in listeriosis outbreaks and sporadic cases. Although the occurrence of *L. monocytogenes* is low, potential virulent *L. monocytogenes* isolates may pose a potential risk to consumers. This study emphasizes the need for continuous surveillance for this pathogen in aquatic products in China.

## Author Contributions

QW, JZ, and MC conceived and designed the experiments. MC, JC, and YC performed the experiments. LX, HZ, SW, and QY conducted bioinformatics analyses. MC, QW, JZ, and TL drafted the manuscript. QW, JB, and JW reviewed the manuscript. All authors read and approved the final manuscript.

## Conflict of Interest Statement

The authors declare that the research was conducted in the absence of any commercial or financial relationships that could be construed as a potential conflict of interest.

## Footnotes

1 http://www.stats.gov.cn/tjsj/ndsj/2017/indexch.htm

2 http://bigsdb.pasteur.fr/listeria/listeria.html
