# Supplementary material for: Occurrence, Antibiotic Resistance, and Population Diversity of Listeria monocytogenes Isolated From Fresh Aquatic Products in China
Source: Front Microbiol. 2018 Sep 19;9:2215. doi: 10.3389/fmicb.2018.02215 (PMC6157410; doi:10.3389/fmicb.2018.02215)
Supplement: Supplementary file 1 [file Table_1.docx]

**Supplementary files**

Table S1. The *Listeria monocytogenes* isolates used in this study

| No. | Isolates | No. | Isolates | No. | Isolates | No. | Isolates |
| --- | --- | --- | --- | --- | --- | --- | --- |
| 1 | 12-1LM | 19 | 1139-1LM | 37 | 1981-1LM | 55 | 3031-1LM |
| 2 | 55-1LM | 20 | 1157-1LM | 38 | 2006-1LM | 56 | 3104-4LM |
| 3 | 85-1LM | 21 | 1207-1LM | 39 | 2026-1LM | 57 | 3177-3LM |
| 4 | 189-1LM | 22 | 1257-1LM | 40 | 2026-2LM | 58 | 3179-1LM |
| 5 | 323-1LM | 23 | 1275-1LM | 41 | 2039-1LM | 59 | 3181-1LM |
| 6 | 389-1LM | 24 | 1324-1LM | 42 | 2056-1LM | 60 | 3306-1LM |
| 7 | 507-1LM | 25 | 1324-2LM | 43 | 2081-1LM | 61 | 3354-1LM |
| 8 | 507-2LM | 26 | 1477-1LM | 44 | 2130-1LM | 62 | 3356-2LM |
| 9 | 556-1LM | 27 | 1582-1LM | 45 | 2154-1LM | 63 | 3707-2LM |
| 10 | 557-1LM | 28 | 1627-1LM | 46 | 2155-2LM | 64 | 3756-1LM |
| 11 | 588-1LM | 29 | 1629-1LM | 47 | 2174-1LM | 65 | 3776-1LM |
| 12 | 589-1LM | 30 | 1755-1LM | 48 | 2327-1LM | 66 | 3778-1LM |
| 13 | 704-1LM | 31 | 1757-1LM | 49 | 2328-1LM | 67 | 3877-1LM |
| 14 | 704-3LM | 32 | 1930-1LM | 50 | 2528-1LM | 68 | 3907-1LM |
| 15 | 889-1LM | 33 | 1931-1LM | 51 | 2707-1LM | 69 | 3930-1LM |
| 16 | 955-1LM | 34 | 1954-1LM | 52 | 2904-1LM | 70 | 3981-3LM |
| 17 | 1090-1LM | 35 | 1956-1LM | 53 | 2929-1LM | 71 | 4256-1LM |
| 18 | 1126-1LM | 36 | 1980-1LM | 54 | 3029-1LM | 72 | 4256-2LM |

Table S2. Primers used for serogroup identification of *Listeria monocytogenes* strains

| Target gene | Forward and reverse primers (5'→3') | Specificity | Annealing temperature (°C) | Size of PCR amplicon (bp) | Reference |
| --- | --- | --- | --- | --- | --- |
| *lmo0737* | AGGGCTTCAAGGACTTACCC | *L. monocytogenes* serovars 1/2a, 3a, 1/2c and 3c | 53 | 691 | (Doumith et al., 2004) |
|  | ACGATTTCTGCTTGCCATTC |  |  |  |  |
| *lmo1118* | AGGGGTCTTAAATCCTGGAA | *L. monocytogenes* serovars 1/2c and 3c | 53 | 906 |  |
|  | CGGCTTGTTCGGCATACTTA |  |  |  |  |
| *ORF2819* | AGCAAAATGCCAAAACTCGT | *L. monocytogenes* serovars 1/2b, 3b, 4b, 4d, 4e and 7 | 53 | 471 |  |
|  | CATCACTAAAGCCTCCCATTG |  |  |  |  |
| *ORF2110* | AGTGGACAATTGATTGGTGAA | *L. monocytogenes* serovars 4b, 4d and 4e | 53 | 597 |  |
|  | CATCCATCCCTTACTTTGGAC |  |  |  |  |
| *prs* | GCTGAAGAGATTGCGAAAGAAG | All *Listeria* species | 53 | 370 |  |
|  | CAAAGAAACCTTGGATTTGCGG |  |  |  |  |
|  | TTTATCCGTACTGAAATTCC |  |  |  |  |

Table S3 The breakpoints of antimicrobial resistance of *Listeria monocytogenes* isolates in the study

| Antibiotic agents | Breakpoints (mm or μg/mL) | | |
| --- | --- | --- | --- |
|  | Susceptible | Intermediate | Resistant |
| Kanamycin | ≥ 18 | 14–17 | ≤ 13 |
| Tetracycline | ≥ 19 | 15–18 | ≤ 14 |
| Doxycycline | ≥ 16 | 13-15 | ≤12 |
| Chloramphenicol | ≥18 | 13-17 | ≤12 |
| Gentamicin | ≥ 15 | 13–14 | ≤ 12 |
| Erythromycin | ≥ 23 | 14-22 | ≤13 |
| Rifampin | ≥20 | 17-19 | ≤16 |
| Sulbactam/ampicillin | ≥15 | 12-14 | ≤11 |
| Vancomycin ^*^ | ≥17 | 15-16 | ≤14 |
| Meropenem | ≥16 | 14-15 | ≤13 |
| Linezolid | ≥21 | - | ≤20 |
| Amoxycillin/clavulanic acid | ≥20 | - | ≤19 |
| Penicillin | ≤2 μg/mL | - | - |
| Ampicillin | ≤2 μg/mL | - | - |
| Sulfamethoxazole with trimethoprim | 29 | - | 29 |

* Breakpoint for *Entercococcus spp*.

Table S4. Primers used for the amplification of virulence factors in *Listeria monocytogenes* strains

| Gene | Primers(5'→3') | Annealing temperature (°C) | Length (bp) | Reference |
| --- | --- | --- | --- | --- |
| *prfA* | CTGTTGGAGCTCTTCTTGGTGAAGCAATCG | 60 | 1060 | (Notermans et al., 1991) |
|  | AGCAACCTCGGTACCATATACTAACTC |  |  |  |
| *inlA* | CGGATGCAGGAGAAAATCC | 55 | 2403 | (Wu et al., 2016) |
|  | CTTTCACACTATCCTCTCC |  |  |  |
| *hly* | GTTAATGAACCTACAAGACCTTCC | 60 | 707 | (Xu et al., 2009) |
|  | ACCGTTCTCCACCATTCCCA |  |  |  |
| *inlB* | GATATTGTGCCACTTTCAGGT | 60 | 367 | (Xu et al., 2009) |
|  | CCTCTTTCAGTGGTTGGGT |  |  |  |
| *actA* | CGCCGCGGAAATTAAAAAAAGA | 60 | 839 | (Suarez et al., 2001) |
|  | ACGAAGGAACCGGGCTGCTAG |  |  |  |
| *iap* | ACAAGCTGCACCTGTTGCAG | 60 | 131 | (Furrer et al., 1991) |
|  | TGACAGCGTGTGTAGTAGCA |  |  |  |
| *inlA* | TAATATAAGTGATATAAGCCCAG | 60 | 606 | (Chen et al., 2009) |
|  | TTTATCCGTACTGAAATTCC |  |  |  |
| *plcA* | CTGCTTGAGCGTTCATGTCTCATCCCCC | 60 | 1484 | (Notermans et al., 1991) |
|  | CATGGGTTTCACTCTCCTTCTAC |  |  |  |
| *plcB* | ATGTGCTTGACCGCAAGTGT | 60 | 436 | (Chen et al., 2014) |
|  | CTTCTCGGTAATCAGCCACC |  |  |  |
| *mpl* | GCTTTGCCGGATTCCTGCG | 60 | 1086 | This study |
|  | CTTCTTATTCGCCCATCTCGCG |  |  |  |
| *llsX* | TTATTGCATCAATTGTTCTAGGG | 52 | 200 | (Clayton et al., 2011) |
|  | CCCCTATAAACATCATGCTAGTG |  |  |  |
| *ptsA* | TCCTTTTTCTTTGTTGCGGA | 52 | 450 | (Maury et al., 2016) |
|  | TCTGAAGCTGTACGAAGACA |  |  |  |

References:

Chen, J., Luo, X., Jiang, L., Jin, P., Wei, W., Liu, D., et al. (2009). Molecular characteristics and virulence potential of *Listeria monocytogenes* isolates from Chinese food systems. Food Microbiol. 26, 103–111. doi: 10.1016/j.fm.2008.08. 003

Chen, M., Wu, Q., Zhang, J., Yan, Z., and Wang, J. (2014). Prevalence and characterization of *Listeria monocytogenes* isolated from retail-level ready-to-eat foods in South China. Food Control 38, 1–7. doi: 10.1016/j.foodcont.2013.09.061

Clayton, E. M., Hill, C., Cotter, P. D., and Ross, R. P. (2011). Real-time PCR assay to differentiate Listeriolysin S-positive and -negative strains of *Listeria monocytogenes*. Appl. Environ. Microbiol. 77, 163–171. doi: 10.1128/AEM. 01673-10

Furrer, B., Candrian, U., Hoefelein, C., and Luethy, J. (1991). Detection and identification of *Listeria monocytogenes* in cooked sausage products and in milk by in vitro amplification of haemolysin gene fragments. J. Appl. Bacteriol. 70, 372–379.

Maury, M. M., Tsai, Y. H., Charlier, C., Touchon, M., Chenal-Francisque, V.,Leclercq, A., et al. (2016). Uncovering *Listeria monocytogenes* hypervirulence by harnessing its biodiversity. Nat. Genet. 48, 308–313. doi: 10.1038/ng.3501

Notermans, S. H., Dufrenne, J., Leimeisterwächter, M., Domann, E., and Chakraborty, T. (1991). Phosphatidylinositol-specific phospholipase C activity as a marker to distinguish between pathogenic and nonpathogenic *Listeria* species. Appl. Environ. Microbiol. 57, 2666–2670.

Suarez, M., Gonzalez-Zorn, B., Vega, Y., Chico-Calero, I., and Vazquez-Boland, J. A. (2001). A role for ActA in epithelial cell invasion by *Listeria monocytogenes*. Cell. Microbiol. 3, 853–864.

Wu, S., Wu, Q., Zhang, J., Chen, M., and Guo, W. (2016). Analysis of multilocus sequence typing and virulence characterization of *Listeria monocytogenes* isolates from Chinese retail ready-to-eat food. Front. Microbiol. 7:168. doi: 10.3389/fmicb.2016.00168

Xu, X., Wu, Q., Zhang, J., Deng, M., and Zhou, Y. (2009). Studies on specific detection of *Listeria monocytogenes* in foods by duplex PCR. Chin. J. Health Lab. Technol. 19, 1199–1201.

Table S5. Primers used for MLST analysis

| Primer | Sequences(5’→3’) | Length (bp) | Tm (°C) |
| --- | --- | --- | --- |
| *abcZoF* | **GTTTTCCCAGTCACGACGTTGTA**TCGCTGCTGCCACTTTTATCCA | 537 | 52 |
| *abcZoR* | **TTGTGAGCGGATAACAATTT**CTCAAGGTCGCCGTTTAGAG |  |  |
| *bglAoF* | **GTTTTCCCAGTCACGACGTTGTA**GCCGACTTTTTATGGGGTGGAG | 399 | 45 |
| *bglAoR* | **TTGTGAGCGGATAACAATTT**CCGATTAAATACGGTGCGGACATA |  |  |
| *catoF* | **GTTTTCCCAGTCACGACGTTGTA**ATTGGCGCATTTTGATAGAGA | 486 | 52 |
| *catoR* | **TTGTGAGCGGATAACAATTT**CAGATTGACGATTCCTGCTTTTG |  |  |
| *dapEoF* | **GTTTTCCCAGTCACGACGTTGTA**CGACTAATGGGCATGAAGAACAAG | 462 | 52 |
| *dapEoR* | **TTGTGAGCGGATAACAATTT**CATCGAACTATGGGCATTTTTACC |  |  |
| *datoF* | **GTTTTCCCAGTCACGACGTTGTA**GAAAGAGAAGATGCCACAGTTGA | 471 | 52 |
| *datoR* | **TTGTGAGCGGATAACAATTT**CTGCGTCCATAATACACCATCTTT |  |  |
| *ldhoF* | **GTTTTCCCAGTCACGACGTTGTA**GTATGATTGACATAGATAAAGA | 453 | 52 |
| *ldhoR* | **TTGTGAGCGGATAACAATTTC**TATAAATGTCGTTCATACCAT |  |  |
| *lhkAoF* | **GTTTTCCCAGTCACGACGTTGTA**AGAATGCCAACGACGAAACC | 480 | 52 |
| *lhkAoR* | **TTGTGAGCGGATAACAATTT**CTGGGAAACATCAGCAATAAAC |  |  |
| *LhkA*-F3 | GCAAGTTTTGAATACGTATCAGCG (Lineage 3) | 480 | 52 |
| *LhkA*-R2 | TACGCATTTCATGAGAAACATCAG (Lineage 3) |  |  |
